# Attitudes of secondary school adolescents towards premarital sexual activity and associated factors in Fako, Cameroon

**DOI:** 10.4314/ahs.v25i1.38

**Published:** 2025-03

**Authors:** Rita Muso Fubam, Oladapo Olayemi, Akin-Tunde Ademola Odukogbe, Nicholas Tendongfor

**Affiliations:** 1 Department of Obstetrics and Gynaecology, Pan African University Life and Earth Sciences Institute, University of Ibadan, Ibadan, Nigeria; 2 Department of Obstetrics and Gynaecology, College of Medicine, University of Ibadan/University College Hospital, Ibadan, Nigeria; 3 Department of Public Health and Hygiene, University of Buea, Buea, Cameroon

**Keywords:** Adolescents, attitude, Cameroon, sexual behaviour, reproductive health

## Abstract

**Background:**

Adolescents continue to engage in risky sexual behaviours. These behaviours can be influenced by their attitude regarding premarital sexual activity.

**Aim:**

This paper aimed to assess attitude of secondary school adolescents towards premarital sexual activity and the associated factors in Fako, Cameroon.

**Method:**

A cross-sectional study was conducted among 1180 adolescents in secondary schools in Fako, Cameroon. A questionnaire was administered to assess their knowledge and attitudes regarding sexual and reproductive health. Descriptive analysis and inferential statistics were used to identify factors associated with their attitude toward premarital sexual activities. p value < 0.05 with 95% confidence interval indicated statistical significance.

**Results:**

More than half (54.6%) of the adolescent students had more liberal attitudes regarding premarital sexual activity. Being male (AOR= 1.931, 95% CI= 1.400, 2.663), discussing sexuality-related information with family (AOR=1.423, 95% CI=1.034-1.958), and using the internet to obtain sexuality-related information (AOR= 1.397, 95% CI: 1.023-1.397) were associated with more liberal attitudes towards premarital sexual activities. Younger adolescents had less liberal attitudes (AOR=0.541, 95% CI= 0.361-0.810).

**Conclusion:**

More liberal attitudes towards premarital sexual activity was reported among adolescents hence stakeholders concerned with adolescent reproductive and sexual health should design strategies, including internet-based interventions that address any negative attitudes about sexuality among adolescents.

## Introduction

Premarital sexual activity among adolescents continues to increase worldwide with young people nowadays having a more open attitude towards sexual activities when compared to the past decades^1^. Increasing trends in age at first marriage also increases the likelihood of premarital sexual activity among adolescents^2^. According to the 2018 Cameroon Demographic Health Survey (CDHS), 18% of women and 7% of men aged between 25 to 49 years began sexual activity by the age of 15 years^3^. Although sexuality is inherent to human existence, its expression can involve some behaviours and activities that are considered risky with a likelihood for adverse health outcomes. Several unfavourable statistics continue to show the consequences of risky sexual behaviours which affect not only the adolescents involved but also their families, communities and nations. A systematic review reported a prevalence of 18.8% adolescent pregnancy in Africa^4^. In Cameroon, the pregnancy rates among adolescent women aged 15 to 19 years has been reported as 24%^3^. There is growing concern of STIs among adolescents; in the United States of America for instance, half the number of new STIs infections annually is reported among young people 15 to 24 years^5^. The consequences of risky sexual behaviours are not limited to physical ones but also psychological and socioeconomic consequences^6^-^8^. These remain major global public health concerns which continue to undermine the Sustainable Development Goals. Attitudes toward sexual activity have been documented as a factor which influences sexual decision making and behaviour^9^-^11^. Other studies have reported associations between sexual attitudes and gender, age, religiosity and sexual and reproductive health knowledge^12^-^14^. Adolescents continue to face a rapidly changing environment and exposure to the media especially social media which most likely contain sexual contents. This has been reported to influence their sexual attitudes^12^. Attitudes and behaviours that are learned and developed in adolescence can extend into adulthood. The statistics earlier mentioned show that even in countries like Cameroon where adolescent premarital sexual activity is not condoned, adolescents still engage in such, a trend also reported in other countries^2^,^9^. There is dearth of studies especially in Cameroon that provide evidence on the attitude of adolescents towards premarital sexual activities. Considering the consequences of risky sexual behaviours among adolescents, it is imperative for researchers and policy makers especially in low-resource settings like Cameroon, to explore the factors that can influence adolescents' perceptions and attitudes regarding premarital sexual behaviours in order to proffer targeted and cost-effective solutions and interventions. This study therefore determined the attitudes of secondary school adolescents towards premarital sexual activity and the factors associated with such attitudes among them in Fako, Cameroon.

## Methods

### Study design

This paper is derived from an institution-based cross-sectional study which is part of a larger study by the authors to conduct a needs assessment for a school-based curriculum for sexual and reproductive health education in Fako, Cameroon.

### Study setting

Fako Division is one of the six and most populated divisions in the Southwest Region of Cameroon. Three of its four sub-divisions were purposively selected as they were considered the most stable during the on-going socio-political crises in the country. Hence, these sub-division are also the predilection sites for internally displaced persons including school-going adolescents from conflict areas in and around the South West Region of Cameroon.

### Study population and sampling

The study was conducted among 1180 adolescent secondary school students in Fako. Those included were students aged 12 to 19 years old. Nine secondary schools were randomly selected from the three subdivisions included in the study. These included public, faith-based and lay private schools. In each school, male and female students were recruited by multistage random sampling techniques. The selection included students from all levels ranging from Form one (6 th grade) to Upper Sixth (12th grade).

### Study variables

The outcome variable was attitude to premarital sexual activity. This was assessed through nine items which addressed to what extent the adolescent considered different sexual behaviours appropriate. These questions were drawn from WHO's Illustrative Core Instruments^15^. A simple 3-point Likert scale (agree, neutral and disagree) was used to assess responses, this took into consideration the comprehension level of the younger adolescents who took part in the study. The measure of attitude towards premarital sexual activities was obtained by calculating a composite score from the nine items. Values were assigned to the responses taking into consideration whether the questions were positively or negatively worded. The maximum score of 18 was obtained and dichotomized at a cut-off point of 10 as the mean score was 9.7. Thus, those who scored 10 and above were categorized as having a more liberal attitude towards premarital sexual activities while those who scored less than 10 were categorized as having less liberal attitude towards premarital sexual activities. Explanatory variables included age, gender, religion, type of school, kind of education, class, living arrangement, family-adolescent discussion on sexuality related matters and internet use to obtain sexuality-related information.

### Data collection

An interviewer-administered questionnaire was used to collect data. Trained interviewers guided the respondents through the questionnaires. This was done at a suitable time that was arranged by the school authorities in order to avoid interrupting learning activities. The interviews were conducted in the English language. Data collection spanned from November 2020 to January 2021. The questionnaire was pretested one week before the actual data collection on five percent of the total same size that was not included in the main survey.

### Data analysis

Univariate analysis including frequencies and percentages were performed on the characteristics of participants and to categorise attitudes to premarital sexual activities. Simple logistic regression was used in bivariate analysis to identify potential predictors of liberal attitudes to premarital sexual activities. Multivariate logistic regression was conducted using variables with p-values< 0.2 to identify factors independently associated with liberal attitudes to premarital sexual activities. Statistical significance was set at p<0.05. Data analysis was done using SPSS version 26.0.

## Ethical consideration

This study had approval from the University of Ibadan/University College Hospital, Ibadan (UI/UCH) Ethical Review Committee with approval number UI/UCH-EC: 017/20. Approval was also obtained from the Southwest Regional Delegation of Secondary Education in Cameroon, followed by permission from the school principals. Once approval was obtained, the research team worked with the teacher assigned to aid the team to obtain consent from the guardians of the adolescent students. Before data collection, assent was obtained from the participants after they were informed about the study objectives, rights to voluntary participation and confidentiality of data.

## Results

### Descriptive characteristics of study participants

The mean age of the 1180 adolescent secondary students who participated in this study was 15.48 + 2.13 years of age with more than half of them (63.8%) being between 15 to 19 years; 56.0% were females; and 65.0% were in the lower secondary school. Majority of the participants were of the Christian faith (92.8%) and attending public schools (62%); more than half were currently living with both parents (55.6%). About three fifths (59.6%) of the students lived in households with more than five persons; 59.5% had sexuality related discussions with their families and less than half (45.4%) had used the internet obtain information related to sexuality (Table 1).

**Table 1:** Sociodemographic characteristics of participants

Variables		Frequency	Percentage (%)
**Age range(years)**	12-14	427	36.2
	15-19	753	63.8
**Mean ± SD**	15.48±2.13		
**Gender**	Male	519	44.0
	Female	661	56.0
**Class category**	Lower secondary	767	65.0
	Upper secondary	413	35.0
**School type**	Public	727	61.6
	Private	233	19.7
	Faith – based	220	18.6
**Religion**	Christian	1095	92.8
	Muslim/others	85	7.2
**Currently living with**	Single parent	345	29.2
	Both parents	651	55.2
	Others	184	15.6
**Sexuality-related discussions with family**	Yes	478	40.5
	No	702	59.5
**Access to Internet**	Yes	774	63.1
	No	436	36.9
**Uses internet for sexuality information**	Yes	338	45.4
	No	406	54.6

### Attitude to premarital sexual activities and associated factors

More than half of the study participants (54.3%) had more liberal attitudes related to premarital sexual activities. As shown in Table 2, bivariate analysis showed that age, gender, class, school type, having sexuality related discussions with family and using the internet to obtain information related to sexuality were potential predictors of more liberal attitudes towards premarital sexual activities among the participants. After adjusting for confounders independent predictors of more liberal attitudes towards premarital sexual activities were age, gender, school type, having sexuality-related discussions with family and using the internet to obtain information related to sexuality, as they remained statistically significant. Compared with older adolescent age group (15-19 years), students in the younger age group (12-14 years) were less likely to have more liberal attitudes toward premarital sexual activities (AOR= 0.54, 95% CI: 0.36, 0.81). Males were about two times more likely to have more liberal attitudes (AOR= 1.93, 95% CI: 1.40, 2.66) when compared with females. Students who were enrolled in lay-private schoolhad higher odds of more liberal attitudes to premarital sexual activity (AOR= 1.95, 95% CI: 1.19-3.19) when compared with those in public and mission schools. The odds of more liberal attitudes among participants who had sexuality-related discussions with family members were higher (AOR= 1.42, 95% CI: 1.03-1.96) when compared with those who did not have such discussions. The use of the internet to obtain information related to sexuality was shown to increase the odds of more sexually liberal attitudes among the participants (AOR= 1.4, 95% CI: 1.02-1.4).

**Table 2:** Factors associated with liberal attitudes to premarital sexual activities among adolescent students in Fako

Variable	Level	OR (95%CI)	p-value	AOR (95%CI)	p-value
**Age (years)**	12-14	0.45 (0.35-0.57	0.000	0.54 (0.36-0.81)	**0.003**
	15-19	1		1	
**Gender**	Male	1.76 (1.39-2.23)	0.000	1.93 (1.40-2.66)	**0.000**
	Female	1			
**Class**	Lower secondary	0.59 (0.46-0.75)	0.000	0.79 (0.55-1.15)	0.221
	Uppersecondary	1		1	
**School type**	Public	1.38 (1.02-1.87)	0.037	1.07 (0.72-1.60)	0.733
	Lay-private	2.21 (1.52-3.22)	0.000	1.95 (1.19-3.19)	**0.008**
	Faith-based	1		1	
**Religion**	Christian	1.17 (0.75-1.82)	0.484		
	Muslim/others	1			
**Currently living with**	Single parent	1.05 (0.73-1.51)	0.784	1.25 (0.77-2.03)	0.378
	Both parents	0.76 (0.55-1.06)	0.105	0.81 (0.52-1.26)	0.345
	Others	1			
**Sex-related discussions with family**	Yes	1.42 (1.13-1.80)	0.003	1.42 (1.03-1.96)	**0.030**
	No	1		1	
**Uses the internet for sex-related information**	Yes	1.64 (1.22-2.21)	0.001	1.4 (1.02-1.4)	**0.036**
	No	1		1	
**Attended SRH class in school**	Yes	0.94 (0.74-1.2)	0.630		
	No	1			

## Discussion

This study determined the attitude to premarital sexual activities and associated factors among adolescents in Fako, Cameroon. Above half of the students had more liberal attitude towards premarital sexual activities. In a similar study, almost half of the students had liberal attitudes toward premarital sexual activities^14^. In another study by Wang and colleagues, 60% of participants had liberal attitudes towards premarital sexual activity^11^. Although there is a general notion that young people should abstain from sexual activities, the finding of this study indicates that many of the young people have liberal attitudes regarding sexual behaviours and activity. This is important as this attitude can lead to young people engaging in premarital sexual activities, which is often risky with many health and life implications. The present study showed that being in the younger adolescent age group was a protective factor against more liberal attitude. This finding is similar with the results of previous research^9^. This finding however contradicts those of another study in which younger adolescents were more likely to have more liberal attitudes when compared to older ones 14. Often, younger adolescents receive different forms of warning about engaging in sexual activities from parents and other older adults. Considering the level of trust younger adolescents have in their parents, they tend to take their caution seriously and this can affect their attitudes towards sexual behaviours as reported in the current study. The effect of peer influences on the younger adolescents may also not be as strong as in the older adolescents who have gained a little more independence from parental control. There is less exposure to environmental cues which can influence the attitudes of younger adolescents than it is for older adolescents who are usually fairly independent. Our findings thus imply that timely development of an appropriate curriculum and its adequate implementation can prevent younger adolescents from the liberal attitudes and sexual experimentation associated with the older adolescents.

The study found that male adolescent students had significantly more liberal attitude regarding premarital sexual behaviours. In most societies including this study setting, there still exist gender differences and restrictions that can influence sexual behaviour and even expression of sexual views in the different genders. There continue to be more rigid expectations with regards to the expression of female sexuality compared to males. In some cultures, male involvement in sexual activity is a sign of sexual competence and a sign that they are mature enough. On the other hand, female virginity is still highly valued and considered a virtue especially in cultures that still maintain conservative norms like in Fako, Cameroon, hence sexual activity in unmarried females is greatly shamed^1^. Similar studies among adolescents have also reported more liberal attitudes among male participants^10^, ^12^, ^14^, ^16^. This finding is of great concern because although males have more liberal attitudes, females are disproportionately affected by negative sexual and reproductive health outcomes.

Discussing sexuality-related matters with family members was found to be independently associated with more liberal attitudes. Adolescents who had discussions with family members on matters related to sexuality were more likely to have more liberal attitudes compared to those who did not. Wang and colleagues reported similar findings in which male and female participants who communicated with father and mother respectively regarding sex related matters had more open attitude toward premarital sexual activities^11^. The present study did not probe into the nature of sexuality related discussions that the participants had with their families. Future in-depth studies should consider the nature and content of sexuality-related discussions between family members especially parent-child discussions. In cultures where sexuality related discussions are still not freely done or conducted with a warning tone, parents may pay attention on warning the adolescents rather than equipping them with how to explore their sexuality in ways that are safe and appropriate.

It will also be necessary to know about the living conditions of the adolescents which may negatively or positively affect the interactions between parents and offspring, even when the contents of the parental teachings are appropriate. In the present study, there was a significant association between using the internet to obtain information related to sexuality and more liberal attitudes. This is comparable to other studies in which more liberal attitudes were reported among adolescent participants who had access to online contents^9^, ^12^. As adolescent exposure to social media increases, which may include exposure to sexually explicit information and images, this may negatively influence the attitude and practices related to risky sexual activities in these adolescents.

## Strengths and limitations

As a strength, this study provides insight into the attitude towards premarital sexual activities of adolescent students in Fako which is lacking even among the general Cameroonian research literature. Secondly, the sample was drawn from among students in different formal education settings giving a more diverse and representative sample of adolescents.

Thirdly, it has revealed that younger adolescents are still generally having a less liberal attitude to premarital sexual activities, giving a window of opportunity to put in place a curriculum and structures to keep them so. However, the study was not without limitations; although a relatively large sample size participated in the study, the findings cannot be generalised to all adolescents in Fako, Cameroon as students in the rural areas and out-of-school adolescents were not included. Further research can be conducted to increase generalizability among all adolescents including out-of-school adolescents. Secondly, the data used in this study were self-reported by the students. Future studies can in addition, make use of qualitative methodology to gain an in-depth understanding of adolescents' attitudes regarding premarital sexual behaviour in this context. Despite these limitations, the findings of this study are foundational in exploring in-depth the sexual attitudes and related factors among Cameroonian adolescents.

## Conclusions and Recommendations

The results of this study show more liberal attitudes regarding premarital sexual behaviour among adolescent secondary school students in Fako, Cameroon. The associated factors being age, gender, type of school, exposure to sexuality related information from family and the internet. The risks of poor sexual health outcomes and their consequences are likely to increase with more liberal attitude to premarital sexual activity among adolescents coupled with the sexual experimentation and inadequate knowledge that often characterise people in this age group. The findings of this study will be made available to sexual and reproductive health program designers, policy makers and other stakeholders concerned with adolescent reproductive and sexual health to enable a deeper understanding of the sexual attitudes of adolescents. These stakeholders can then design strategies and interventions that seek to address negative attitudes about sexuality that are potentially damaging to the health of the adolescents. Evidence – based, age-appropriate sexuality education should be provided to these adolescents in schools to empower them with appropriate knowledge and skills. Considering their influences, families and internet program designers should be actively involved in developing and implementing strategies or interventions that target the reproductive health of adolescents. Adolescents should be provided knowledge and skills on safe use of the internet.
